# SARS-CoV-2-specific immune responses in elderly and immunosuppressed participants and patients with hematologic disease or checkpoint inhibition in solid tumors: study protocol of the prospective, observational CoCo immune study

**DOI:** 10.1186/s12879-022-07347-w

**Published:** 2022-04-25

**Authors:** Alexandra Dopfer-Jablonka, Sandra Steffens, Frank Müller, Marie Mikuteit, Jacqueline Niewolik, Anne Cossmann, Metodi V. Stankov, Georg M. N. Behrens, Eva Hummers, Gloria Heesen, Dominik Schröder, Sascha Roder, Frank Klawonn, Kai Vahldiek, Justin Hasenkamp, Jonathan Kallusky, Christine S. Falk, Tobias R. Overbeck, Stephanie Heinemann

**Affiliations:** 1 Department for Rheumatology and Immunology, Hannover Medical School, Hannover, Germany; 2 German Center for Infection Research (DZIF), Partner Site Hannover-Braunschweig, Hannover, Germany; 3 CiiM-Centre for Individualised Infection Medicine, Hannover, Germany; 4 Department of General Practice, University Medical Center Göttingen, Humboldtallee 38, 37073 Göttingen, Germany; 5 Department of Computer Science, Ostfalia University of Applied Sciences, Wolfenbuettel, Germany; 6 Clinic for Hematology and Medical Oncology, University Medical Center Göttingen, Göttingen, Germany; 7 Institute of Transplant Immunology, Hannover Medical School, Hannover, Germany

**Keywords:** SARS-CoV-2, COVID-19, Pandemic, Humoral and cellular immunity, Immunocompromised people, Elderly, Hematology, Solid tumor, Checkpoint inhibition, Serological testing, Coronavirus, Social participation, Immunogenicity

## Abstract

**Background:**

Immunocompromised people (ICP) and elderly individuals (older than 80 years) are at increased risk for severe coronavirus infections. To protect against serious infection with SARS-CoV-2, ICP are taking precautions that may include a reduction of social contacts and participation in activities which they normally enjoy. Furthermore, for these people, there is an uncertainty regarding the effectiveness of the vaccination. The COVID-19 Contact (CoCo) Immune study strives to characterize the immune response to COVID-19 vaccination in immunocompromised, elderly people, and patients with hematological or oncological diseases. The study uses blood-based screenings to monitor the humoral and cellular immune response in these groups after vaccination. Questionnaires and qualitative interviews are used to describe the level of social participation.

**Methods:**

The CoCo Immune Study is a mixed methods prospective, longitudinal, observational study at two large university hospitals in Northern Germany. Starting in March 2021, it monitors anti-SARS-CoV-2 immune responses and collects information on social participation in more than 600 participants, at least 18 years old. Inclusion criteria and subcohorts: Participants with (1) regularly intake of immunosuppressive medication (ICP-cohort) or (2) age ≥ 80 years (80 + -cohort). Additionally, patients with current or former (3) myeloid, (4) lymphatic disease or (5) solid tumor under checkpoint inhibition (3–5: HO-cohort). Exclusion criteria: (1) refusal to give informed consent, (2) contraindication to blood testing, (3) inability to declare consent. Participants complete a questionnaire at four different time points: prior to full vaccination, and 1, 6 and 12 months after completed vaccination. In addition, participants draw blood samples themselves or through a local health care provider and send them with their questionnaires per post at the respective time points after vaccination. Patients of the HO cohort dispense additional blood samples at week 3 to 12 and at month 6 to 9 after 2nd vaccination to gain additional knowledge in B and T cell responses. Selected participants are invited to qualitative interviews about social participation.

**Discussion:**

This observational study is designed to gain insight into the immune response of people with weakened immune systems and to find out how social participation is affected after COVID-19 vaccination.

*Trial registration:* This study was registered with German Clinical Trial Registry (registration number: DRKS00023972) on 30th December 2020.

**Supplementary Information:**

The online version contains supplementary material available at 10.1186/s12879-022-07347-w.

## Background

In late 2019, the severe acute respiratory syndrome coronavirus 2 (SARS-CoV-2) emerged in Wuhan, China, and has spread throughout the world [1]. To date, over 90,000 persons (about 0.1% of the population) in Germany have died due to an infection with SARS-CoV-2 [2]. Elderly individuals as well as people with pre-existing conditions are at increased risk for severe or fatal infections [3, 4]. Of particular interest are immunocompromised people (ICPs), e.g. patients under immunosuppression drug therapy due to autoimmune disease such as multiple sclerosis, inflammatory bowel disease, rheumatologic and dermatologic autoimmune disorders as well as solid tumor patients treated with immunotherapy and patients with myeloid or lymphatic disease and patients with hematopoietic stem cell transplantation, CAR-T cell therapy or solid organ transplantation. Recent studies showed that solid organ transplant recipients and patients suffering of maligne hematologic diseases have severely increased risk of dying [5–9]. However, this has not been revealed for many rheumatology patients with immunosuppressive therapy, except those who receive higher doses of corticosteroids [10].

The European Medicines Agency approved two nucleoside-modified mRNA vaccines (BNT162b2 (BioNTech, Mainz, Germany) & mRNA-1273 (Moderna, Cambridge, USA, Massachusetts)) and two recombinant replication-incompetent chimpanzee adenovirus vector (AZD1222 (Oxford-AstraZeneca, Cambridge, United Kingdom) & Ad26.COV2.S (Johnson & Johnson, New Brunswig, USA, New Jersey) COVID-19 vaccines that are currently used within the European Union. All of these vaccines currently show good to outstanding efficacy varying depending on the virus variant in preventing deaths and transmissibility [11]. Little is known about the acute and long term immune response after COVID-19 immunization in elderly people and ICP [12]. A reduced production of neutralizing antibodies after vaccination against pneumococcus and influenza has been described in immunosuppressive drugs that reduce humoral defense such as Rituximab and Methotrexate [13–15]. Two recent studies showed that functional humoral immunity to a single respective two doses Pfizer-BioNTech is impaired by Methotrexate but not by targeted biologics, whereas cellular responses are preserved [16, 17]. A study from England shows that Adalimumab and Infliximab impair humoral vaccine response [18]. However, data on longer terms with more patients is still lacking.

In addition to clarify these immediate biomedical knowledge needs, there is additional lack of understanding of how ICP and elderly people experience the vaccine era. To protect against serious infection with SARS-CoV-2, many ICP and elderly persons are taking precautions that may include a reduction of social contacts and reduced participation in activities which they normally enjoy. Furthermore, for these people, there is an uncertainty regarding the effectiveness of the vaccination. After receiving the COVID-19 vaccine, it is often assumed that ICP and elderly may be more inclined to resume social contacts and regular daily activities.

The main objectives of the COVID-19 Contact (CoCo) Immune Study are therefore to determine both, (a) if a COVID-19 vaccination provokes humoral immunity to the SARS-CoV-2 spike glycoprotein, defined as neutralizing antibody responses to wild-type SARS-CoV-2, and spike-specific T cell responses and (b) if and how elderly people and ICP return to usual activities and participation in social aspects of life after vaccination. The study uses serological and T cell screenings to monitor the immune response in participants after vaccination as well as questionnaires and qualitative interviews to assess the level and kinds of social participation.

An important aspect of carrying out a study during the pandemic is the reduction of physical contact throughout the study. Not only do participants in clinical studies normally have personal contact to study personnel during the recruitment and information process, but also during data collection as well. Study participants may use public transportation and most likely will spend time in clinic buildings and waiting rooms, etc. Such study-related situations may unnecessarily increase the risk of SARS-CoV-2 infection and also introduce a selection bias.

Because the study participants of the CoCo Immune Study are especially vulnerable for infections, special organizational and ethical challenges need to be addressed. One way of keeping infection risks as minimal as possible, is to offer participants the option of a self-administered capillary blood sampling at home (for which there are detailed step-by-step instructions) instead of a venous blood draw at a practice or hospital. The blood samples will be sent per post using addressed, pre-paid small cardboard packages. The feasibility of self-administered capillary blood sampling is therefore another aspect of the study which will be evaluated, in order to make recommendations for later projects.

## Methods

### Aims and setting

In March 2020, the Department for Rheumatology and Immunology at the Hannover Medical School initiated the CoCo study to address central questions regarding the risk of COVID-19 in healthcare personnel and the utility of serological screenings for SARS-CoV-2 in healthcare professionals [19].

In March 2021, based on the experience and results obtained during the CoCo study, the “CoCo Immune Study” was developed in collaboration with colleagues from the University Medical Center Göttingen, with the aim of focusing on regular serological screenings in elderly persons, ICP and HO as well as the mixed-methods exploration of social participation in these groups. The CoCo Immune Study has the following primary and secondary objectives:

Primary objectives

- to systematically assess the humoral immune reactions (e.g. anti-SARS-CoV-2 Spike IgG antibodies) in ICP and elderly persons early following full COVID-19 vaccination
- to assess changes in the participants’ self-perceived level of social participation over time (measuring from before full vaccination up until 12 months following vaccination)

Secondary objectives

- to longitudinally characterize the humoral immune response after COVID-19 and influenca vaccinations of ICP and elderly persons (e.g. magnitude and persistence)
- to estimate if age, chronic diseases or immunosuppressive therapy have an effect on the body’s immune reaction to vaccination
- to assess cellular response in HO cohort
- to find out if ICP are pausing immunosuppressive therapy before or between vaccination doses in hopes of increasing the immune reaction to vaccination
- to assess the rate of individuals interrupting immunosuppressive therapy during vaccination
- to describe the experiences of immunocompromised persons during the pandemic including (mental) health and wellness, social participation
- to understand study participants’ views regarding the COVID-19 vaccination
- to assess the feasibility of capillary blood sampling procedures by the study participants themselves (as a measure for reducing the risk of exposition to SARS-CoV-2 infection associated with a visit to a doctor’s office or clinic for a venal blood draw)

### Sample size

The CoCo Immune Study has a mixed methods study design, combining serological screening, standardized questionnaires as well as qualitative interviews to gain a comprehensive understanding of the health and social situation of elderly persons, ICP and HO before and after COVID vaccination. This study is explorative in nature and does not seek to test hypotheses. Therefore, the results of this study may provide the basis for a power calculation for future studies.

*Quantitative study* 50 individuals per group minimally are expected to be sufficient to reliably describe humoral immune responses after vaccination. We have the financial capability to process the blood tests for 800 study participants and plan to recruit them as follows:

ICP cohort:

- Persons with other forms of immunsuppression (≥ 2.5 mg Prednisone, anti-IL-6, Anti-IL-1, etc.) (n = 250)

80 + cohort:

- Persons ≥ 80 years (n = 75)

HO cohort:

- B-CLL or multiple myeloma (n = 120)
- B-Cell-Depletion due to Rituximab (incl. Biosimilars or Obinutuzumab) (n = 50)
- Therapy with immune checkpoint inhibitors (anti-PD-1, PD-L1, CTLA-4) (n = 50)
- Patients after stem cell transplantation or CART therapy (n = 50)

*Qualitative study* We will focus upon the experiences of the ICP cohort only. A size of 20 subjects is expected to be sufficient to work out an overview of typical case histories and experiences.

### Inclusion and exclusion criteria

*Quantitative study. Inclusion criteria*

Participants, at least 18 years old, with (1) regular intake of an immunosuppressive medication or (2) age ≥ 80 years. Further, patients with current or former (3) myeloid or (4) lymphatic disease w/o anti-CD20 directed therapy or (5) solid tumor under checkpoint inhibition (HO) AND full immunization against SARS CoV-2 not have occurred more than 30 days prior to study enrollment (counted from the first vaccination for Johnson & Johnson vaccine or the second vaccination for all other vaccinations).

Immunsuppressive medication is defined as regularly intake of Prednisone (≥ 2.5 mg/d), Methotrexate, Etanercept, Vedolizumab, Leflunomid, Tacrolimus / Everolimus / Sirolimus, Adalimumab, Fingolimod, Rituximab, Mycophenolatmofetil, Secukinumab, Certolizumab, Dimethylfumarat, Upadacitinib, Ustekinumab, Hydroxychloroquin, Dasatinib, Ixekizumab, Tocilizumab, Glatirameracetat, Apremilast, Azathioprin, Ocrelizumab, Ciclosporin, Golilumab, Infliximab, Baricitinib, Natalizumab, Interferone, Ibrutinib, Obinutuzumab, Abatacept or combinations of the aforementioned drugs.

*Exclusion criteria* (1) refusal to give informed consent, (2) contraindication to blood testing, (3) inability to declare consent.

*Qualitative study* Interviewees will be a sub-sample of the above-described CoCo Immune Study participants. We will use the demographic data to consider gender, age, education, urban/rural location and underlying health conditions, aiming at maximum variability. Only those persons who have agreed to be re-contacted for further research questions will be approached for an interview. To prevent selection bias, additional ICPs may also be recruited who have not previously participated in the cohort study.

### Characteristics of participants and recruitment

Study participants will be informed about study participation by newspaper announcements, homepage and social media posts, posters at vaccination centers, local general practices and clinics for patients requiring immunosuppressive therapy or 80 years and older throughout the Northern German region of Lower Saxony. Further recruiting will take place in the outpatient clinics of the Department of Rheumatology and Immunology at the Hannover Medical School and the Department of Hematology and Oncology at the University Medical Center Göttingen.

### Description of all processes, interventions, comparisons

**Fig. 1:**
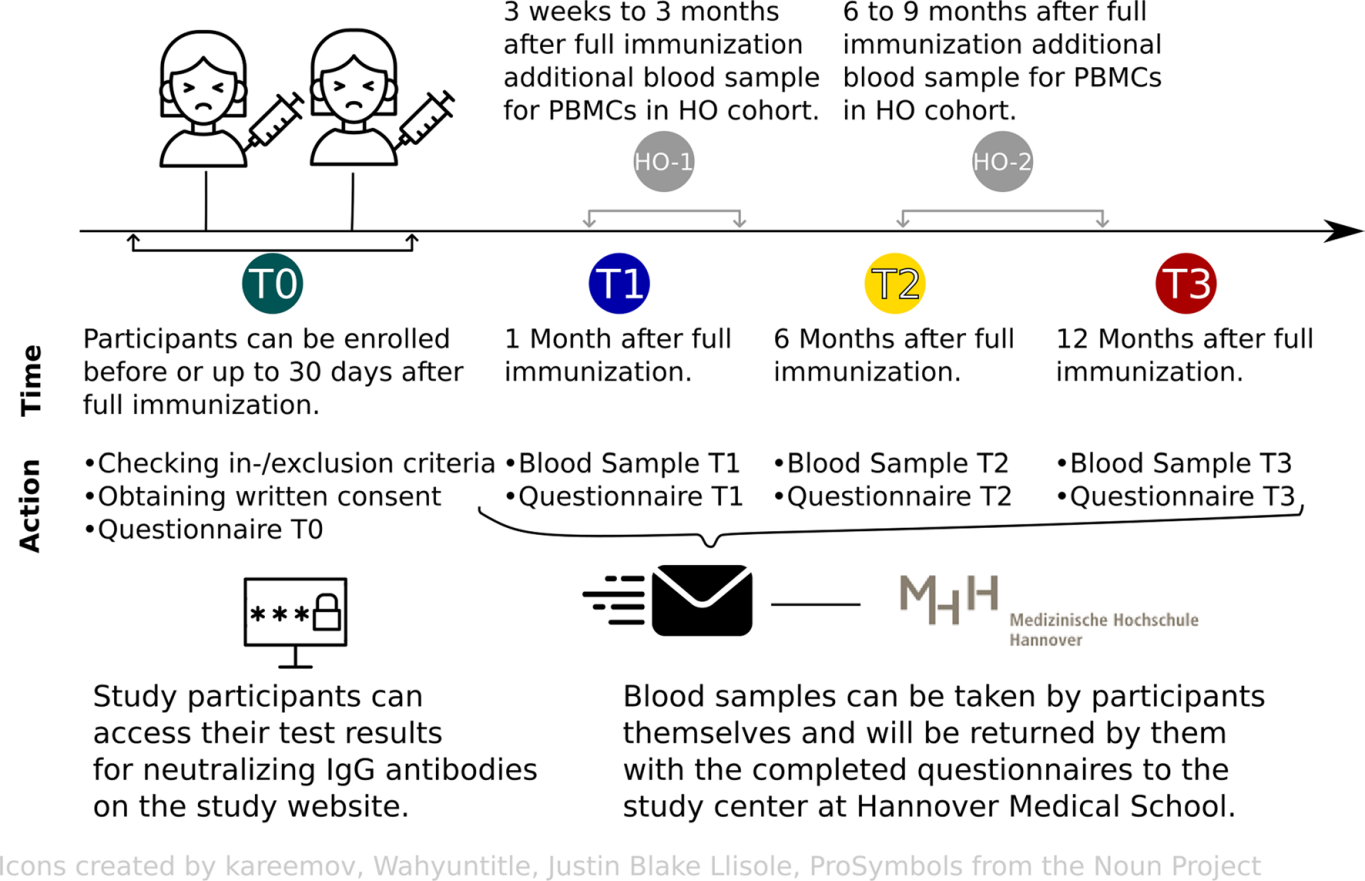
Process of the CoCo Immune Study

**Fig. 2:**
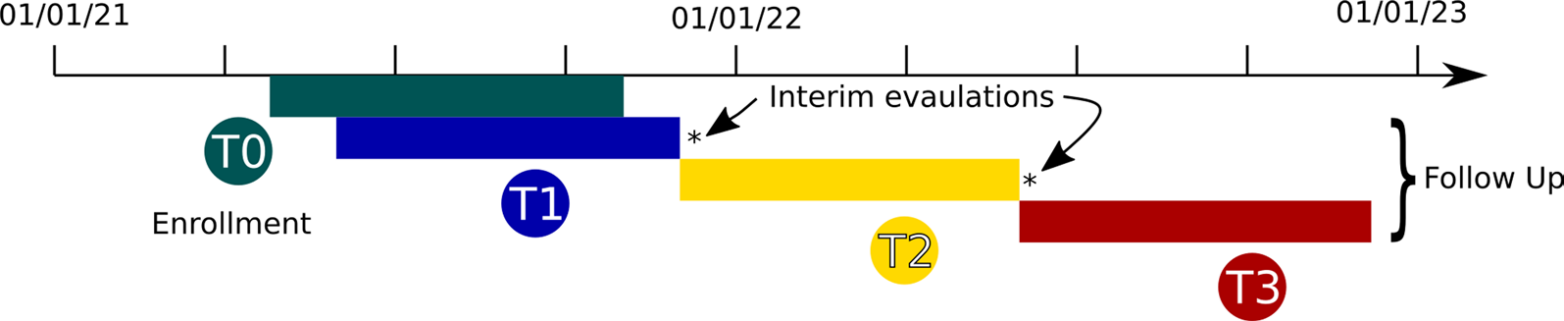
Timeline of the study

*Quantitative study* After written informed consent is obtained, all participants will be asked to fill out a baseline questionnaire at enrollment (T0), preferably before completion of full vaccination. At 1 month (T1), 6 months (T2) and 12 months (T3) following full vaccination (see Fig. 1), all study participants will be asked to fill in follow-up questionnaires and give blood samples, which will be returned to the study center in Hannover by mail. Blood can be drawn by the study participants themselves (as capillary blood in 500 μl EDTA tubes), members of the research team, or in doctor’s office or clinics during routine visits (2.6 ml, 7.5 ml or 500 µl EDTA tubes). Patients of the HO cohort dispense additional blood samples at week 3 to 12 (HO-1) and at month 6 to 9 (HO-2) after 2nd vaccination for analysis of cellular immune responses (Fig. 2).

No incentives for participation in the quantitative part of the CoCo Immune Study will be offered, but participants will be given access to the results of their blood tests via web-based personalized access codes.

*Qualitative study* Participants will be contacted by telephone and/or email, informed and asked to participate in the interview study. Interviewees will be offered 40€ compensation for their time and travel costs.

### Data collection: what outcomes will be measured, when and how

*Quantitative study* The variables collected are listed in Table 1. At baseline, study participants will be asked to provide information about their age, gender and health status (e.g. presence of cardiovascular or respiratory conditions, regular medication, etc.) as well as their attitudes about vaccinations in general and the COVID-19 vaccine in particular. Furthermore, social participation and wellbeing will be measured. All questionnaires are paper-based and processed using EvaSys (EvaSys GmbH, Lüneburg, Germany). Additional information on therapy or diseases, such as the detailed stages of tumor diseases, can also be extracted from medical records where applicable.

**Table 1:** Schedule of enrolment and assessments

	Study period				
	Enrolment	Allocation	Post-allocation		
Timepoint**	*-t*_*1*_	0	*1 M*	*6 M*	*12 M*
*Enrollment*					
Eligibility screen	X				
Informed consent	X				
Allocation		X			
*Assessments*					
General information (date of enrollment, demographic data e.g. age, sex, educational level, residential and housing status, care level, disabilities, migration status)	X	X			
Drug therapy / intake (diagnoses, pre-existing conditions, smoking status, previous SARS-CoV-2 infections, immunosuppressive or immunomodulating drug therapy and if they have been paused during vaccination (T1))		X	(X)		
Vaccination status (timing of COVID-19 vaccinations, number of vaccinations, name of applied COVID-19 vaccines)			X		
Serum / Plasma (ELISA / multiplex analyses for e.g. anti-SARS-COV-2 S1 specific IgG/IgA)			X	X	X
PBMCs (Analysis of cellular immune responses against SARS-CoV-2 in HO cohort)			X (HO-1)	X (HO-2)	
Social Participation and general wellbeing (Index for the Assessment of Health Impairments (IMET)) [20]; additional self developed items to measure social participations and overall wellbeing in the pandemic situation; PHQ-4 Questionnaire [21, 22])		X	X	X	X
Attitudes towards vaccination (Questionnaire items from the Preventable Infectious Disease Survey from the German Federal Center for Health Education [23], additional self developed items specific to measure COVID-19 attitudes ex-ante (T0) and post-vaccination (T1), experience with previous vaccinations (e.g. side effects)		X	X		
Experience with self-administered capillary blood taking (willingness to draw blood independently, prior experience with medication injections or blood glucose monitoring (T0), whether independent blood collection was successful or help (doctors, pharmacies) was sought (T1–T3))		X	X	X	X
Qualitative Interviews (semi-structured interviews on social participation)			––––––––––––––		

*Qualitative study* Semi-structured interviews will last about 45–60 min. All conducted interviews will be transcribed according to the simplified rules of Kuckartz [24] and Dresing/Pehl [25] and subsequently analyzed using qualitative content analysis according to Mayring [26] and Kuckartz [24].

### Data management plans

A data management plan was created for the project based on the recommendations of the Digital Curation Center [27]. This document can be found as an appendix to this article (see Additional file 1). Briefly, data will be published in anonymized form after completion of the analysis and publication of the aforementioned research questions. Research data will be published via the Research Data Repository of the University of Göttingen and will be assigned a DOI. Samples will be transferred to the biobank of the Hannover Medical School and will be available for further research under given data protection, organizational and ethical guidelines.

### Safety considerations

The study is designed as an observational study without any intervention and therefore low-risk for participants. Due to the current pandemic situation and in order to protect the subjects and study personnel, the entire study will be conducted with minimal contact. Participants who are interested can obtain information about participation in advance on the internet. In addition, participants may contact the study team via video conference or telephone in case of questions. All necessary study materials including sets for capillary blood sampling can also be shipped to participants. All participants received verbal and written instruction about collecting a capillary blood sample, including a single page with step-by-step illustrations. The samples are then returned per mail to the study center at Hannover Medical School and analyzed in the laboratory of the Department of Rheumatology and Immunology. Sample handling and processing is performed by the Hannover Medical School. Interviews will be conducted either in person or as a videoconference, depending upon the pandemic situation and/or preferences of the interviewees.

### Type of data and statistical analyses planned

The data will be prepared in SPSS (IBM, Armonk, NY), data analysis performed in R. Visualizations will be created using GraphPad Prism (GraphPad Software, San Diego, CA) and R.

Statistical analyses comprise:

1. Analysis of blood samples for SARS-CoV-2 antibodies at T1, T2 and T3 to assess which immunoglobulins are produced following vaccination and how long they persist in ICP and elderly persons. Estimates and confidence intervals for the mean, median and selected lower quantiles will be computed. For the comparison of time points, the t-, Wilcoxon-Mann–Whitney and the Kolmogorov–Smirnov test will be applied including corrections for multiple testing.
2. Analysis of self-perceived level of social activity over time (T0, T1, T2 and T3) using standardized questionnaires. Scores derived from questionnaires will be analysed w.r.t. to change over time based on the t- and the Wilcoxon-Mann–Whitney test including corrections for multiple testing.
3. Analysis of ICP who paused therapy including the recommendation to pause therapy. A group comparison between persons who paused and did not pause therapy will be carried out using the t- and the Wilcoxon-Mann–Whitney with a possible stratification w.r.t. covariates like age, health state and medication. Logistic regression will be applied to identify factors that are associated with pausing therapy.
4. Analysis of the feasibility of self-performed capillary blood sampling procedures at T1, T2, T3. It will be analysed whether certain factors like age or education have an influence on the feasibility of self-performed capillary blood sampling procedures for the corresponding persons. The t-, the Wilcoxon-Mann–Whitney and Fisher’s exact test will be applied.
5. Analysis of study participants’ views regarding vaccinations in general and the COVID vaccination specifically at T0. Differences between groups, e.g. gender, age, education health status and medication, will be investigated based on the t-, the Wilcoxon-Mann–Whitney and Fisher’s exact test. Logistic and ordinal regression will be applied to identify factors that influence the attitude to COVID vaccination.

### Laboratory setup and immunological analyses

All samples will either be collected in outpatient clinics and express-mailed directly to the lab or collected by self-administered capillary blood collection and mailed to the lab by regular post. The research lab is located at Hannover Medical School and all samples will be processed and stored on the day of arrival at the lab. Serological testing for anti-Spike IgG will be performed on all samples. On samples of the HO cohort, additional tests, such as cellular immune responses will be performed (Table 2). Hannover Medical School is a collaborating center of regional and national networks, such as the German Center for Infection Research. Some analyses can be performed at other partner sites, if necessary.

**Table 2:** Planned analyses

Type of specimen	Planned analyses
Serum/plasma	•ELISA for SARS-COV-2 S1 specific IgG/IgA •Multiplex analyses of SARS-COV-2 S1, S2, RBD •ELISA for Influenza A/B specific IgG
PBMC	•SARS-CoV-2 Interferon Gamma Release Assay (IGRA) in HO subgroups

### Serological testing

As a primary screening system, a quantitative ELISA for anti-SARS-CoV-2 spike protein 1 (S1) immunoglobulin G (IgG) will be used. We will use the CE certified version of the Anti-SARS-CoV-2-QuantiVac-ELISA (IgG) from Euroimmun, Lübeck, Germany. A semi-quantitative ELISA detecting anti-SARS-COV-2 S1 immunoglobulin A (IgA) will be performed in a subset of samples. In order to discriminate IgG specific for the spike regions S1, S2, and RBD multiplex analyses for IgG will be performed with plasma samples (1:200 dilutions) using the Luminex-based multiplex analyses (MiIliplex HC19SERG1-85). For Interferon Gamma Release Assays (IGRA) the Quant-T-Cell SARS-CoV-2 (Euroimmun, Lübeck, Germany) kits will be used.

Furthermore, influenza A/B IgG will be measured in selected frozen samples (i.e. those negative to anti-SARS-CoV-2 IgG). A suitable testing system will be selected when all samples have been acquired.

All remaining plasma samples will be available for additional research questions, in case novel serology testing systems (e.g. for assessing viral entry inhibition) or experimental tools evaluating neutralization of novel SARS-CoV-2 variants of concern become available.

### Ethical considerations and declarations

The study is registered at the German Clinical Trial Register (DRKS00023972) and approved by the Institutional Review Board of both Hannover Medical School (Approval No. 8973_BO_K_2020) and University Medical Center Göttingen (Approval No. 29/3/21). A data security management plan has also been approved. Written informed consent will be obtained from all study participants. Each participant will receive written information on the study procedures and data management. Participants will be informed about specimen and data collection, as well as storage of samples for future research projects. Study participation is voluntary and participants have the right to withdraw consent at any time and without disclosure of reasons for withdrawal. Furthermore, participants receive a written data security protocol. A trained member of the study team will be available for questions at enrollment or at any later time point of time during the study.

### The status and timeline of the study

*Quantitative study* The study began in March 2021 with the first recruited participant. Recruitment is currently ongoing. The last participant in is planned for November 2021.

*Qualitative study* During the T0-T1 time frames, the semi-structured interview guideline will be developed and tested. The recruitment of interview participants, data collection and data analysis will take place during T2 and T3.

## Discussion

This study aims at analyzing broader effects of the SARS-CoV-2 vaccination. This clinical trial is designed to gain insight into the antibody response of people with weakened immune systems and to find out how much social participation is affected by vaccination.

A limitation of our study is that vaccine efficacy is measured indirectly by serology and we do not systematically test probands for SARS-CoV-2 infection by PCR or other techniques. We furthermore focus on humoral immune responses and cannot comprehensively assess cellular or local immune responses, e.g. in the upper airways. Obviously, protective immunity against SARS-CoV-2 cannot be ascertained by serological testing analyses alone, but should be complemented by virus inhibition assays, the assessment of further adaptive and innate cellular and humoral factors and the analysis of infection rates. However, the objective of this study is not to assess protection but to characterize functional immune responses.

This study will report on serological data and social participation in relation to factors such as time, age, gender, immunosuppression. The mixed-methods approach will also include deeper insights into the experiences of ICP. A short description of the study design will be included in each published manuscript and this protocol will be referenced. Participant and public involvement will be encouraged by the study website and by personal contact to the study team by phone. Participants will be encouraged to obtain their study results and give feedback on the study design, especially regarding the feasibility of the self-administered blood draw.

Due to the rapidly changing circumstances during the pandemic, the study protocol will be amended as needed. All changes will be notified to the institutional review board.

## Supplementary Information

**Additional file 1:** Data Management Plan.

## Data Availability

Not applicable.

## Abbreviations

CoCo: COVID-19 contact
COVID-19: Coronavirus disease 2019
HO: Haematological oncology
ICP: Immunocompromised people
IgA: Immunoglobulin A
IgG: Immunoglobulin G
IGRA: Interferon gamma release assays
SARS-CoV-2: Severe acute respiratory syndrome coronavirus 2
S1: SARS-CoV-2 Spike 1 protein
