# Supplementary material for: SARS-CoV-2-specific immune responses in elderly and immunosuppressed participants and patients with hematologic disease or checkpoint inhibition in solid tumors: study protocol of the prospective, observational CoCo immune study
Source: BMC Infect Dis. 2022 Apr 25;22:403. doi: 10.1186/s12879-022-07347-w (PMC9035970; doi:10.1186/s12879-022-07347-w)
Supplement: Supplementary file 1 — Additional file 1: Data Management Plan. [file 12879_2022_7347_MOESM1_ESM.docx]

Data Management Plan

for

*CoCo Immune Study*

# Project description

**What is the main research question of the project?**

The CoCo Immune study strives to characterize the immune response to COVID-19 vaccination in ICP and elderly people. The study uses serological screenings and questionnaires to monitor the immune response in ICP after vaccination as well as the level of return to usual activities and participation in social aspects of life.

**Which persons or institutions are responsible for the project coordination?**

- Hannover Medical School, PD. Dr. Alexandra Jablonka

**Which persons or institutions are responsible for the data management in the project?**

- Hannover Medical School, Prof. Georg Behrens
- University Medical Center Göttingen, Frank Müller MD Msc

# Project policies

**Does your institution have rules or guidelines for the handling of research data? If yes, please briefly outline them and refer to more detailed sources of information if necessary. Please also indicate, if the rules / guidelines are mandatory or optional.**

none

**Does the funder have rules or recommendations for data management? If yes, please briefly outline them and refer to more detailed sources of information if necessary. Please also indicate, if the rules / guidelines are mandatory or optional.**

none

**Are there requirements regarding the data management from other parties (e.g. the scholarly/scientific community)?**

none

# Data Collection - What data will you collect or create?

**What kind of data is it?**

*Questionnaire T0:* Questionnaire survey containing data on participants' date of enrollment; demographic data (e.g. age, sex, educational level, residential and housing status, care level, disabilities, migration background), Index for the Assessment of Health Impairments (IMET); additional self developed items to measure social participations and overall wellbeing; PHQ-4 Questionnaire; Diagnoses, pre-existing conditions, smoking status, previous COVID-Infections, Immunosuppressive or immunomodulating drug therapy; Questionnaire Items from the Preventable Infectious Disease Survey from the German Federal Center for Health Education, additional self developed items specific to measure COVID-19 attitudes ex ante; Preparedness to draw blood independently, Prior experience with medication injections or blood glucose monitoring

*Questionnaire T1:* Index for the Assessment of Health Impairments (IMET ); additional self developed items to measure social participations and overall wellbeing; PHQ-4 Questionnaire; additional self developed items specific to measure COVID-19 attitudes post vaccination; whether independent blood collection was successful or help (doctors, pharmacies) was sought.

*Questionnaire T2 & T3:* Index for the Assessment of Health Impairments (IMET ); additional self developed items to measure social participations and overall wellbeing; PHQ-4 Questionnaire; whether independent blood collection was successful or help (doctors, pharmacies) was sought

*Labdata #1:* ELISAs for IgG specific for the SARS-CoV-2 spike glycoprotein, date of analyses, comments on analyses

**Is the data being created or re-used?**

*Questionnaire T0:* Created

*Questionnaire T1:* Created

*Questionnaire T2 & T3:* Created

*Labdata #1:* Created

**If re-used, under which address, PID or URL can the dataset be found?**

*n/a*

**If re-used, who created the dataset?**

*n/a*

**Which file formats are used?**

*Questionnaire T0:* CSV

*Questionnaire T1:* CSV

*Questionnaire T2 & T3:* CSV

*Labdata #1:* CSV

**What is the actual or expected size of the dataset?**

*Questionnaire T0:* less than 1 GB

*Questionnaire T1:* less than 1 GB

*Questionnaire T2 & T3:* less than 1 GB

*Labdata #1:* less than 1 GB

# How will the data be collected or created?

**Which tools, software, technologies or processes are used to generate or collect the data?**

Questionnaire instruments are described above. Additional items will be described in an upcoming article. As a primary screening system, a semiquantitative ELISA for SARS-COV-2 spike protein 1 (S1) IgG is used (Euroimmun, Lübeck, Germany – CE certified version is used.

**Are there internal project guidelines for a consistent organisation of the data? If so, where they are documented?**

No

**Is there a internal project guideline for naming the data? If so, please briefly outline the naming conventions and, if necessary, link to the documentation.**

No

**Which measures of quality assurance are taken for the data?**

Review of entered data in Questionnaires. Use of unique non-consecutive pseudonyms. Data cleansing takes place via SPSS syntax and remains transparent throughout all steps.

# How will you handle versioning?

**Are different versions of the dataset created?**

*Questionnaire T0:* No

*Questionnaire T1:* No

*Questionnaire T2 & T3:* No

*Labdata #1:* No

**Which versioning strategy is applied for this dataset?**

*Questionnaire T0:*

*Questionnaire T1:*

*Questionnaire T2 & T3:*

*Labdata #1:*

**Which technology or tool is used for versioning?**

*Questionnaire T0:*

*Questionnaire T1:*

*Questionnaire T2 & T3:*

*Labdata #1:*

# Documentation and Metadata - What documentation and metadata will accompany the data?

**Which information is necessary for other parties to understand the data (that is, to understand their collection or creation, analysis, and research results obtained on its basis) and to re-use it?**

Codebook of the questionnaires, Study protocol.

**Which standards, ontologies, classifications etc. are used to describe the data and context information?**

- It has not yet been decided, with which system the metadata and contextual information will be described

**Who is responsible for documenting the metadata and context information and for checking if they are correct and complete?**

- Frank Müller, MD

# Ethics and Legal Compliance

**Does the data contain personal data?**

Yes

**Will the data be anonymised or pseudonymised?**

Yes, during the collection

**To what extent is the "informed consent" obtained from the persons concerned?**

For analysis / use of the data within the project as well as for re-use.

**Under which terms of use or license will the dataset be published or shared?**

- Attribution (BY)
- Non-commercial (NC)
- Share-alike (SA)

**Do other intellectual property rights apply to this dataset?**

*none*

**Was investigated who the rights owner is?**

*No*

# Storage and Backup - How will the data be stored and backed up during the research?

**Where is the dataset stored during the project?**

*Questionnaire T0:* On local PCs of the research group.

*Questionnaire T1:* On local PCs of the research group.

*Questionnaire T2 & T3:* On local PCs of the research group.

*Labdata #1:* On local PCs of the research group.

*Blood samples:* Physical bloodsample tubes are stored in the biobank of Hannover Medical School.

**How and how often will backups of the data be created?**

*Questionnaire T0:* Backups are made on a daily basis.

*Questionnaire T1:* Backups are made on a daily basis.

*Questionnaire T2 & T3:* Backups are made on a daily basis.

*Labdata #1:* Backups are made on a daily basis.

*Blood samples:* not applicable

**Who is responsible for the backups?**

Questionaire data:

- Frank Müller, MD

*Labdata:*

- Alexandra Jablonka, MD

How will you manage access and security?

**What are the risks to data security and how will these be managed?**

In the event of a possible data loss, the risk is low due to the pseudonymization and the associated separate storage of the key lists. Biobank could be robbed, but that has never happened before.

**Which measures or provisions are in place to ensure data security (e.g. protection against unauthorized access, data recovery, transfer of sensitive data)?**

Project implementation is carried out under the aegis of data economy. For example, many characteristics that allow clear identification of subjects, such as date of birth, place of birth or address, are not even collected. Paper-based consent forms, which are stored separately from the study data to be evaluated, serve as key lists. All sample and questionnaire processing is pseudonymized. Informed consent documents always remain with the study center enrolling the patient. Subjects can access serological results via a secure platform using their access code and a password. The handover of this takes place personally, which corresponds to a strong authentication.

# Selection and Preservation - Which data should be retained, shared, and/or preserved?

**What are the criteria / rules for the selection of the data to be archived (after the end of the project)?**

Data are selected that have been analyzed and published on the issues we are prioritizing.

**Which individuals, groups or institutions could be interested in re-using this dataset? What are possible scenarios?**

Data are useful for clinical researchers in various scenarios.

**Shall there be an embargo period before the data are made available?**

After publication of own results.

**What data must be retained/destroyed for contractual, legal, or regulatory purposes?**

Only data in anonymized form is provided. Key lists are destroyed after a lockout period.

What is the long-term preservation plan for the dataset?

**Where will the data (including metadata, documentation and, if applicable, relevant code) be stored or archived after the end of the project?**

*Questionnaire T0:*

- Own institution
- Has not yet been decided

*Questionnaire T1:*

- Own institution
- Has not yet been decided

*Questionnaire T2 & T3:*

- Own institution
- Has not yet been decided

*Labdata #1:*

- Own institution
- Has not yet been decided

*Blood samples:*

- Discipline specific data center: Biobank Hannover Medical School

**How long will the data be stored?**

*Questionnaire T0:* 10 years

*Questionnaire T1:* 10 years

*Questionnaire T2 & T3:* 10 years

*Labdata #1:* 10 years

*Blood samples:* 10 years

**What are the personnel costs associated with long-term preservation for the project?**

*Questionnaire T0:* 0.2 PM

*Questionnaire T1:* 0.2 PM

*Questionnaire T2 & T3:* 0.2 PM

*Labdata #1:* 0.3 PM

*Blood samples:* 1.4 PM

**What is the amount of non-personnel-costs regarding long-term preservation for the project?**

*Questionnaire T0:* 1000 EUR Euro

*Questionnaire T1:* 2000 EUR Euro

*Questionnaire T2 & T3:* 1000 EUR Euro

*Labdata #1:* 1000 EUR Euro

*Blood samples:* 20.000 EUR Euro

# Data Sharing - How will you share the data?

**Will this dataset be published or shared?**

*Questionnaire T0:* Yes, externally limited with individual approval

*Questionnaire T1:* Yes, externally limited with individual approval

*Questionnaire T2 & T3:* Yes, externally limited with individual approval

*Labdata #1:* Yes, externally limited with individual approval

*Blood samples:* Yes, externally limited with individual approval

**If no, please explain why not. Please differentiate between legal and contractual reasons and voluntary restrictions.**

*n/a*

**If yes, how will potential users find out about your data?**

*Questionnaire T0:* Digital ressource within journal articles, Research Data Repository of University of Göttingen (including DOI)

*Questionnaire T1:* Digital ressource within journal articles, Research Data Repository of University of Göttingen (including DOI)0

*Questionnaire T2 & T3:* Digital ressource within journal articles, Research Data Repository of University of Göttingen (including DOI)

*Labdata #1:* Digital ressource within journal articles, Research Data Repository of University of Göttingen (including DOI)

**When will the data be published (if they are)?**

*Questionnaire T0:* planned for Dec. 1, 2022

*Questionnaire T1:* planned for Dec. 1, 2022

*Questionnaire T2 & T3:* planned for Dec. 1, 2022

*Labdata #1:* planned for Dec. 1, 2022

*Blood samples:* planned for Dec. 1, 2022

# Will you pursue getting a persistent identifier for your data?

**Will persistent identifiers (PIDs) be used for this data set?**

*Questionnaire T0:* Yes

*Questionnaire T1:* Yes

*Questionnaire T2 & T3:* Yes

*Labdata #1:* Yes

*Blood samples:* n/a

**Which system of persistent identifiers shall be used?**

*Questionnaire T0:* Handle / DOI

*Questionnaire T1:* Handle / DOI

*Questionnaire T2 & T3:* Handle / DOI

*Labdata #1:* Handle / DOI

*Blood samples:* n/a

**Who is responsible for the maintenance of the PIDs and the object maintenance (i.e. who is responsible notifying the PID-Service about object relocation and the new address)?**

*Questionnaire T0:*

- Göttingen Research Data Repository, University of Göttingen

*Questionnaire T1:*

- Göttingen Research Data Repository, University of Göttingen

*Questionnaire T2 & T3:*

- Göttingen Research Data Repository, University of Göttingen

*Labdata #1:*

- Göttingen Research Data Repository, University of Göttingen

*Blood samples:*

- n/a

# Are any restrictions on data sharing required?

**If there are any restrictions on the re-use of this dataset, please explain why.**

*Questionnaire T0:* unknown

*Questionnaire T1:* unknown

*Questionnaire T2 & T3:* unknown

*Labdata #1:* unknown

*Blood samples:* Inquiry to the Hannover Biobank, Ethics Vote, Cooperation agreement etc.

# Responsibilities and Resources - Who will be responsible for data management?

**Who is responsible for implementing the DMP, and ensuring it is reviewed and revised?**

- Frank Müller, MD

**Will data ownership and responsibilities for RDM be part of any consortium agreement or contract agreed between partners?**

All institutions involved in the project have signed a data processing contract and a cooperation agreement.

# What resources will you require to deliver your plan?

**How will the datamanagement costs of the project be covered?**

Included in third-party funding of the project.

**To what extent will infrastructure resources be required (e.g. CPU hours, bandwidth, storage space... etc.).**

Infrastructure resources of the usual workplace equipment are sufficient.

**Are there actual or potential usage scenarios that could benefit from support by a data management or IT expert, or that even require such support?**

No
